# Potential Impacts of Climate Warming on Water Supply Reliability in the Tuolumne and Merced River Basins, California

**DOI:** 10.1371/journal.pone.0084946

**Published:** 2014-01-20

**Authors:** Michael Kiparsky, Brian Joyce, David Purkey, Charles Young

**Affiliations:** 1 Wheeler Institute for Water Law & Policy, University of California, Berkeley, California, United States of America; 2 Stockholm Environment Institute, Davis, California, United States of America; University of Oxford, United Kingdom

## Abstract

We present an integrated hydrology/water operations simulation model of the Tuolumne and Merced River Basins, California, using the Water Evaluation and Planning (WEAP) platform. The model represents hydrology as well as water operations, which together influence water supplied for agricultural, urban, and environmental uses. The model is developed for impacts assessment using scenarios for climate change and other drivers of water system behavior. In this paper, we describe the model structure, its representation of historical streamflow, agricultural and urban water demands, and water operations. We describe projected impacts of climate change on hydrology and water supply to the major irrigation districts in the area, using uniform 2°C, 4°C, and 6°C increases applied to climate inputs from the calibration period. Consistent with other studies, we find that the timing of hydrology shifts earlier in the water year in response to temperature warming (5–21 days). The integrated agricultural model responds with increased water demands 2°C (1.4–2.0%), 4°C (2.8–3.9%), and 6°C (4.2–5.8%). In this sensitivity analysis, the combination of altered hydrology and increased demands results in decreased reliability of surface water supplied for agricultural purposes, with modeled quantity-based reliability metrics decreasing from a range of 0.84–0.90 under historical conditions to 0.75–0.79 under 6°C warming scenario.

## Introduction

There is a near consensus among scientists that the Earth's climate is changing, and that under even the best-case scenarios of emissions and climate sensitivity, climate impacts are virtually certain [1], [2]. Climate change is a global environmental problem, but humans will be most concerned with the local and regional effects. One of the most robust findings in climate impacts research is that climate change will alter hydrology and water resources around the globe. In California, two decades of studies of projected climatic impacts on water systems have progressed from hydrologic systems, to agricultural systems, to water storage and conveyance systems [3]. Impacts on hydrology will cascade directly into human and ecological systems at all scales. In California, as in other snow dominated watersheds, climate change will result in reduced snowpack storage, reduced streamflow, and changing seasonal flow patterns that will challenge the resilience of coupled water, energy, agricultural, and ecological systems [4], [5].

To the extent that scenarios of future water supply reliability model water deliveries, such modeling has often been derived from historical climate [6] that may neither accurately represent past [7] nor future [3], [8] climatic conditions. This disconnect has motivated interest in formal integration of climate change into water planning, and the uncertainties inherent in both water systems modeling and climate modeling suggest that incorporating climate impacts on hydrology and water resources could help define potential anticipatory responses.

Projected hydrologic impacts of climate change have been the subject of many studies, and assessment of the potential impacts on water rights holders and environmental flows are starting to appear in the literature. Vulnerability assessments can use ‘outcome oriented’ approaches [9] to describe potential impacts and adaptation options quantitatively by integrating results from multiple models. This can be done using a cascade of modeling information, from large-scale, coarse-grained models, to finer resolution models that cover less spatial or conceptual area but represent specific processes of interest in more detail.

**Figure 1:**
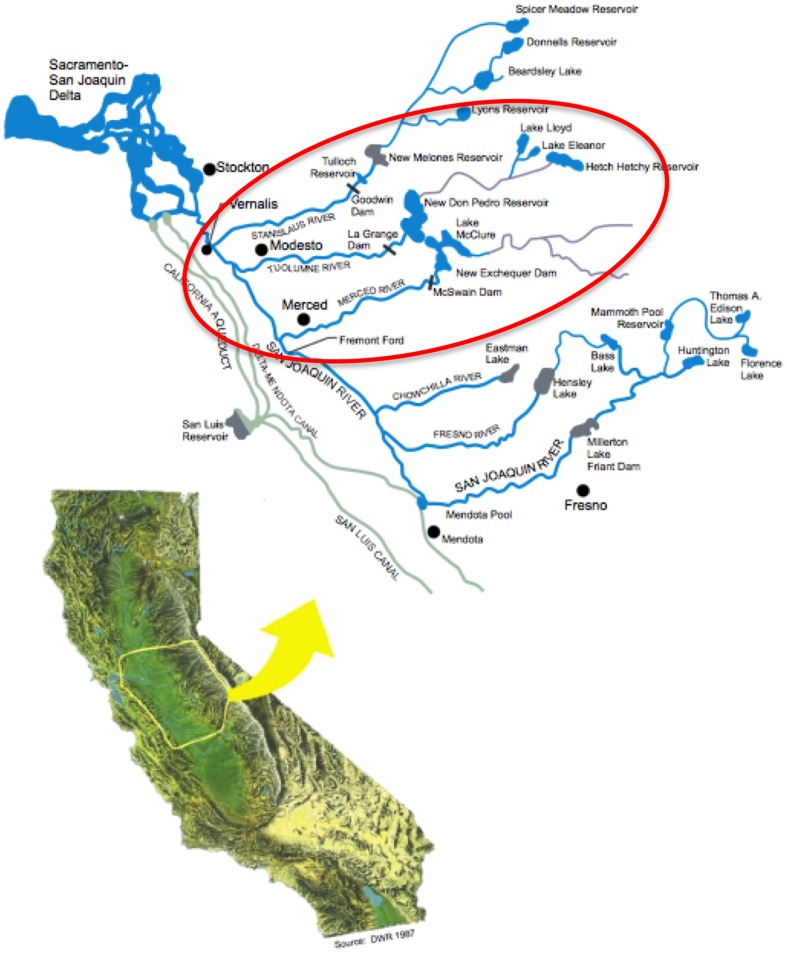
Map of project area for case study. The Stanislaus, Tuolumne, and Merced Rivers flow from the Sierra Nevada Mountains to the San Joaquin River, and thence north towards the Sacramento-San Joaquin Delta, the “hub” of California's water supply system. Figure source: [73].

This paper describes the development of a modeling tool and its application to a sensitivity analysis for temperature warming in the Merced and Tuolumne River Basins in California's Central Valley (Figure 1). To evaluate impacts on hydrology and water supply reliability, we used the Water Evaluation and Planning (WEAP) [10], [11] as a framework to model hydrology and water operations in the three case study basins.

This paper presents the model structure and calibration procedure for the WEAP model of these three basins, along with results from a sensitivity analysis for temperature increases of 2°C, 4°C and 6°C. The model represents seasonal and inter-annual historical hydrologic variability, as well as variation in reservoir levels and surface water deliveries to the major irrigation districts (IDs) in the region. Our findings show hydrologic impacts of temperature change in the form of shifts in timing of streamflow and increased evapotranspiration demands for irrigation water. When interacting with the modeled representation of the managed water system, the impacts manifest as decreased water supply reliability and lower reservoir storage volumes. Thus, while system attenuation of the climate change signal may occur through the capacity of large reservoir and conveyance systems to buffer altered hydrology, with modeled operation regimes unchanged, temperature-driven warming and its influence on modeled hydrology translates into decreased surface water supply reliability in these basins. These results suggest avenues for further work with more detailed analysis of climate and other future stressors for water supply in these basins.

## Study Area

As elsewhere, water is important in California economically, politically, and socially. California has been well-studied with respect to future climatic impacts on water [3]. Hydrology in California, as in many mountainous regions, is dominated by the dynamics of snow accumulation and melting. Gleick (1987) demonstrated the sensitivity of this hydrology to climate warming, projecting earlier and higher hydrograph peaks under climate warming scenarios. This general conclusion has proven robust after two decades and dozens of peer-reviewed studies [3], and exemplifies a hydrologic response of the type that threatens snowmelt-dominated hydrologic systems providing water supply to one-sixth of the world's population [4].

The state has ongoing conflict over water, even without the perturbation of climate change. However, California has traditionally been a national leader in the development of innovative environmental policies [12], and climate change is no exception [13], [14]. Recently, legal motivation to consider climate change in planning decisions has increased in the state, primarily on mitigation, but increasingly on adaptation [15], resulting in path-breaking studies and efforts to apply cutting-edge science. Efforts to integrate research into actual anticipatory changes to water management, however, may require more in-depth studies on scales relevant to local decision-makers.

The Tuolumne and Merced River Basins (TM) in California's Central Valley (Figure 1) lie on the western slope of the Sierra Nevada Mountains, where hydrology will be sensitive to climate change [16], [17], [18], [19]. The terminal reservoirs on each system (New Don Pedro Reservoir and Lake McClure, respectively) each have total storage capacity approximately equal to one year of average annual flow. Agricultural diversions dominate use in both basins, with domestic, municipal, and industrial use a minor fraction. Downstream flow in both cases goes through the San Joaquin River to the Sacramento-San Joaquin Delta. Water allocation in these basins is run through a variety of institutions, much of which revolves around legal and regulatory constraints (e.g., water rights and water quality regulations). Notably, regulatory water quality requirements at Vernalis are key drivers of water releases. Decisions made by water organizations such as federal and state agencies, and local IDs are key to system operation.

With some bounding assumptions, and the inclusion of elements of the Stanislaus River system not described in detail here, the basins can be modeled as a distinct hydrologic unit.

## Methods

### WEAP model structure

The WEAP model consists of interlinked modules for both physical hydrology and operations to calculate demands and allocate water at each time step, and has been described in detail elsewhere [10], [20]. A description of a related application to the hydrology of the Sierra Nevada Mountains has been previously described [21], from which the hydrology module described below is derived. We give a brief overview of the model structure and algorithms in this section, and refer the interested reader to these publications for more details.

WEAP's physical hydrology water balance representation consists of several components designed to represent variability in the key hydrologic components relevant to a study at this temporal and spatial resolution. A one-dimensional soil water accounting scheme routes moisture through two soil layers, with empirical functions describing evapotranspiration, surface runoff, sub-surface runoff, and deep percolation.

WEAP's snowmelt model computes effective liquid water input in each time step as the sum of rain plus snowmelt. To get the latter term, snow water equivalent and snow melt are computed using a temperature index snow accumulation model. Assigned melting and freezing thresholds are used to determine a melting coefficient that specifies snowmelt based on available melting energy and the latent heat of fusion. Available melting energy is a function of net solar radiation and a lumped term comprising other available forms of energy that is adjusted during calibration.

Within each catchment, a water balance is computed based on each unique combination of soil and land cover using a continuous mass balance equation. Evapotranspiration from each fractional area is computed using the Penman-Montieth reference crop potential evapotranspiration equation, using crop/plant coefficients assigned to each land cover type. Surface runoff is calculated using a term scaled by a runoff resistance factor that represents surface characteristics such as roughness, Leaf and Stem Area Index, average slope, porosity, etc. In the two-layer soil moisture scheme, interflow and deep percolation are adjusted using a conductivity parameter, which represents an estimate of upper storage conductivity, and a tuning parameter that partitions flow between horizontal and vertical. Alluvial aquifers are represented in the valley portion of the model, and in these catchments the deep water storage layer is removed and the deep percolation term replaced by percolation from the upper layer directly to the aquifer.

Climate inputs affecting evapotranspiration include temperature, relative humidity, wind speed, and insolation (a function of latitude, Julian day, and cloud cover). Catchments containing irrigated agriculture use the upper soil water store as a trigger for irrigation demands. Threshold values assigned to each crop model irrigation efficiency by determining the level of soil moisture reduction that triggers an irrigation demand.

The WEAP model uses a preference- and priority-driven logic to determine allocation of water to agricultural, urban, and in-stream demands. A node-and-link structure connects sources and supplies. Within each time step (monthly in this implementation), a linear program satisfies demands first to nodes with highest priority, then sequentially allocates water to lower priority users until either demands are satisfied or specified constraints preclude further allocation of water. Each demand node may be supplied by multiple water sources. Water supply preferences can be assigned to simulate user behavior when multiple sources are available, such as in a case where surface water is preferred to groundwater.

Reservoirs are simulated based on their physical characteristics as well as operation parameters that reflect decisions based on balancing flood control, water supply, and carryover storage. A *conservation zone* reflects required space for flood control. A *buffer zone* specifies reservoir levels below which releases are limited in each time step to a specified percentage of the existing water in the reservoir. This approach reduces the complex conditional logic by which actual operational decisions are made to an analogue for conservatism of reservoir operators.

The dynamically interconnected model integrates a rainfall-runoff model with infrastructure and operations logic. There are conceptual and operational differences between the modeled representations of the upper watersheds (areas above the large dam on each river) and the valley floor (agriculturally dominated areas below these dams) that reflect the differences between the two areas. In the upper watersheds, land use is predominantly native vegetation, while in the valley floor agriculture dominates and urban centers are larger. In the upper watersheds, terrain is complex, with individual watersheds spanning large elevation ranges, while the lower watersheds are relatively homogenous.

These differences result in different emphasis in the modeling of the upper and lower watersheds within the model. The upper watersheds are modeled with the primarily goal of representing inflows to the major reservoirs in the system, and the sensitivity of those inflows to future changes in climate. The lower watersheds are modeled primarily to represent agricultural and urban demands, the storage and conveyance facilities that deliver water to satisfy those demands, flows of water thought the managed part of the system, and the sensitivity of both demands and deliveries to changes in climate and other variables in future projections. We describe below the component parts of these overlapping and integrated analyses.

### Data and model implementation

This section describes data used for initial parameterization and calibration of the model, and the following section describes calibration procedures. The model relies heavily on previously published work, with some modifications. The hydrology component of the present model has been adapted for the current study to build on previous work in several ways: it has a monthly time step, larger elevation bands, and a different sub-watershed structure than the version we presented previously [21], [22]. Similarly, the agricultural component relies on methods developed for nearby areas of California [20], [23], and we refer the user to these previous reports for more information.

#### Watershed characteristics

We developed a GIS model of the physical and institutional aspects of the study basins to enable representation of spatially explicit watershed characteristics. The study area was first divided geographically at nested scales based on topography using ArcGIS [24]. *Watersheds* were defined hydrologically by the major dam on each of the three rivers that drain the Sierra mountains. Each watershed was further divided into *sub-watersheds* based on *pour points* at which streamflow is simulated (e.g. locations of gages with historical data). Each sub-watershed is further divided into 500 m elevation bands (*catchments*), coarser than Young et al. [21] for computational efficiency. The total number of catchments in the upper watersheds was condensed from 248 to 80, resulting in a range of effective catchment areas from about 2–600 km^2^. Parameters were adjusted to retain seasonal and annual hydrologic variability as described below. Sub-watersheds and catchments were defined in the area below the upper watersheds and bounded by the San Joaquin River, here by institutional boundaries rather than topography. Sub-watersheds define hydrologically distinct units that enable calibration through comparison of known hydrologic response of smaller model sections to modeled response, as well as describing areas meaningful to managers.

Land surface and subsurface characteristics were derived from United States Geological Survey (USGS) digital elevation models [25] at 10 m (upper watersheds) and 30 m (valley floor), and the ArcHydro toolkit [26] was used to delineate the network of streams and rivers. Each sub-watershed in the valley floor is represented as single elevation catchment.

Within each catchment, we defined classifications of land use, land cover, and soil type, and determined the fractional area of each combination. Vegetation and landcover estimates for the upper, mostly non-agricultural watersheds were based on the National Land Cover Dataset (NLCD) [27] (Table S1 in File S1). Land cover, including cropping patterns, for the agriculturally intensive valley floor were based on the California Land and Water Use survey [28], mapped onto 15 categories (Table S2 in File S1). Intersecting cropping patterns with irrigation district locations [29], [30], [31] resulted in estimated percentages of each land cover and crop type. Historical or future changes in cropping patterns over time were not simulated here. Soils were classified based on the SSURGO and STATSGO datasets [32], [33], as described in Young et al. [21].

Dams [34], canals and other conveyances [35] streamflow gage data, and locations [36] were also incorporated, with physical characteristics taken from published sources (Table S3 in File S1). Reservoir evaporation rate is based on available historical average monthly values [37]. Volume-elevation curves used to approximate surface area were derived from data available from the California Data Exchange Center [38] and the United States Geological Survey [39], and Merced ID storage and rating tables. There is no substantial water infrastructure above the New Exchequer Dam on the Merced River. On the Tuolumne River infrastructure exists for water supply and hydropower purposes above the terminal reservoirs. We simplified the schematic and operational regime in each of these basins, and used generalized representations of water storage and release for hydropower production and water diversions outside each basin based on available historical data. Water infrastructure in the Tuolumne River Basin is operated for local irrigation districts and the San Francisco Public Utilities Commission (SFPUC) to satisfy demands for irrigation and urban uses in the San Francisco Bay Area, governed by the Raker Act [40] and we approximate the daily flow-based logic of this agreement with adjusted monthly parameters for joint reservoir storage and an external monthly demand function for San Francisco based on historical and ‘typical’ monthly flows in the San Joaquin Pipeline. Don Pedro Reservoir is represented as two objects to depict the ‘virtual’ storage by SFPUC in that reservoir.

The Sierra crest forms the upper boundary of the three main watersheds in the model. All three rivers flow into the San Joaquin River (SJR), which in turn flows north to the Sacramento-San Joaquin Delta. Because of upstream diversions, these three basins form a somewhat isolated hydrologic unit: the “section of the SJR between Gravelly Ford and Mendota Pool, a reach of approximately 27 km, is generally dry except when releases are made from Friant Dam for flood control” [41]. Thus, we assume Gravelly Ford constitutes an upper boundary of the model, while acknowledging that future policies may alter this assumption. The lower boundaries of each basin are at the confluences of each river with the San Joaquin. Future work could connect this model with a representation of the Sacramento-San Joaquin Delta and west side tributaries to the San Joaquin River. Groundwater basins are based on California Department of Water Resources (DWR) Bulletin 118 [42]. Future work could incorporate more detailed groundwater modeling.

#### Demand centers

The modeling effort described here focuses on the major irrigation districts [29], [30], [31] and urban areas. We classified adjacent areas of land that receive minimal or no surface water supply as separate from the major irrigation districts, although there are technically irrigation districts within the adjacent land area. Contracts for water supply are represented as lower priority transmission links to invoke WEAP's priority-driven allocation logic as described in Section 0.

Don Pedro Reservoir on the Tuolumne River supplies water to Turlock ID and Modesto ID. Surface water from the Merced River supplies water to the Merced Irrigation District and other entities within its Sphere of Influence. Contract water transfers to these ‘non-district’ areas are represented through a low-priority transmission link. The Merced National Wildlife Refuge also receives surface water through the Merced Main Canal, at a priority higher than Merced ID supplies.

#### Historical climate

A 1/8° gridded observed meteorological historical climate dataset [43] was used for historical climate inputs. For each catchment, we selected the gridpoint closest to the catchment centroid, and used the corresponding time series for temperature, precipitation, and average daily wind speed adjusted for the model's monthly time step. To account for possible bias in upper elevation watersheds, we adjusted the 1/8° inputs to actual elevation of each catchment centroid using a lapse rate of 6.5°C per 1000 m elevation change, altering the temperature input for each catchment by the difference between the midpoint elevation of each catchment and the elevation of the corresponding climate input grid point, multiplied by lapse rate. Overall, these adjustments resulted in a slight average decrease in modeled temperature inputs.

As the goal of this study focuses on streamflow into the terminal reservoirs, and because making selective fine-scale adjustments to precipitation estimates would introduce additional uncertainty and bias onto modeled and/or downscaled projections, we left precipitation inputs unmodified. Note that this approach is in effect the same as most other studies of this type that focus calibration efforts on terminal streamflow without examining or reporting sub-watershed bias. In this, as in other mountainous areas, daily precipitation totals can vary greatly between measurement instruments located within a basin [44]. This can be reflected in hydrologic analysis where isolated, but significant, precipitation events are not captured by an existing precipitation measurement network. For example, detailed studies found stream responses that could not be explained by precipitation measurements alone [44]. Similar effects from bias in input climate data have been observed previously [21], [45], [46].

Seasonal wind speed patterns were based roughly on monthly averages for two years of data at the Merced CIMIS station that overlap with the calibration period. For the valley floor, average relative humidity was interpolated between a peak of 90% in January and a low of 45% in July. For the upper watersheds, relative humidity was estimated from DAYMET, a model that generates estimates of historical weather parameters in complex terrain [47], interpolating between an average high humidity of 60% in January and an average low humidity of 23% in September.

#### Urban demands and supplies

We represented urban demands by aggregating urban population projections based on agricultural district boundaries and representing each as a lumped demand node. To do so, we clipped spatially explicit population projection grids [48] to catchment node areas in the valley floor (Table S4 in File S1).

Urban demands are modeled by multiplying estimated population in a given urban node by an estimated per capita water use level. Per capita water use estimates are taken as the ‘baseline’ 1995–2005 values for the San Joaquin Valley from the State Water Resources Control Board's 20x2020 efforts [49], as 939 liters per capita per day. Consumptive use in urban areas was assumed to be 30%. Urban supplies are mostly met by groundwater, or through arrangements with Irrigation Districts for surface supplies.

#### Other water uses

In-stream flows and hydropower releases are modeled based on internally consistent heuristics. Environmental flow requirements are conditional on modeled representations of year type and/or snowpack-based forecasts (Tables S5–S11 in File S1). Delta flow requirements downstream of the model boundary are based on a proxy for flows released to meet water quality requirements [50], [51]. We detail logic for instream flows for each river basin in Material S1 in File S1. We simulated summer hydropower releases in each stream based on approximate historical summer flows and calibrating to observed flows and reservoir levels.

Groundwater use often occurs within irrigation districts in the region even when surface water supplies are seemingly plentiful. To simulate this, we first assigned a supply preference to each agricultural catchment for surface water, then constrained the total amount of demands that can be supplied by surface water to a percentage that reflects estimates [52] of groundwater use in the region. This in effect forces a minimum amount of groundwater pumping even when sufficient surface water is available, and allows increased groundwater pumping to meet demands when surface water deliveries are constrained by hydrology or operations. In areas with no surface water supply, or limited surface water supply, groundwater use accounts for all demands, and no limits on groundwater use are currently modeled.

Groundwater usage is based on unconstrained access to groundwater resources, given other priorities and preferences specified in the model. The model represents groundwater simply as a stock. The model draws from this stock to satisfy demands in accordance with defined preference and priorities, and recharges it based on hydrologic conditions. Groundwater is represented based on sub-basins of the San Joaquin Valley Groundwater Basin, as defined by the California Department of Water Resources [53].

Our representation of groundwater use is within the range suggested by other studies. Table 1 shows average groundwater use within the primary irrigation districts, compared to the range suggested by previous studies. Note that we used data from a water balance conducted by DWR and USBR [52] to parameterize the current model. Both our approach and the DWR water balance partition canal flows between deliveries and system losses (Table 2), calibrating to other known historical values such as gauged surface water flows. Actual groundwater use and system losses are unknown, and should be treated as estimates.

**Table 1:** Groundwater use by district (10^6^ m^3^).

Model node	Modeled groundwater use			Estimated historical minimum pumping	
	Average	Min	Max	Low	High
**Modesto ID**	107	47	190	48	80
**Turlock ID**	296	183	444	195	234
**Merced ID**	249	159	376	10*	226*

Note that range of groundwater use minimums include the sum of district and non-district pumping, except in the case of Merced ID, which includes district pumping only. Sources: [52], [59].

**Table 2:** Parameters for groundwater/surface water allocation of supply to Districts.

Watershed	Model Node	System losses, %	SW constraint, % of total demand	Canal evaporation, %	Max surface water diversion, m^3^/s (cfs)
**TUO**	**Modesto Main**	38	85	0	
**TUO**	**Turlock Main**	30	75	0	
**MER**	**Merced ID N**	33	75	2	2.8 (100)
**MER**	**Merced ID Main**	27	70 before 1991, 60 after	2	56.6 (2000)

#### Other model elements

In the present analysis, we represent as static some system elements that will clearly change in future, including land use/land cover, urbanization, cropping patterns, and institutional elements. Thus, the modeling presented here can be considered a sensitivity analysis for the climatic variable of temperature [54], and future work will report on efforts to incorporate variability in these elements.

Amount and timing of water rights are represented coarsely. If the total diversions to the main canals in the Merced Basin over the course of a water year exceed total annual water rights, no more surface water diversions are allowed for the rest of the water year. Timing of all diversions is limited to the current irrigation months of April to September, per water rights and historical patterns of diversion, except where water is delivered through the Merced Main Canal to the Merced National Wildlife Refuge during some winter months.

### Representation of historical hydrology and water operations

**Figure 2:**
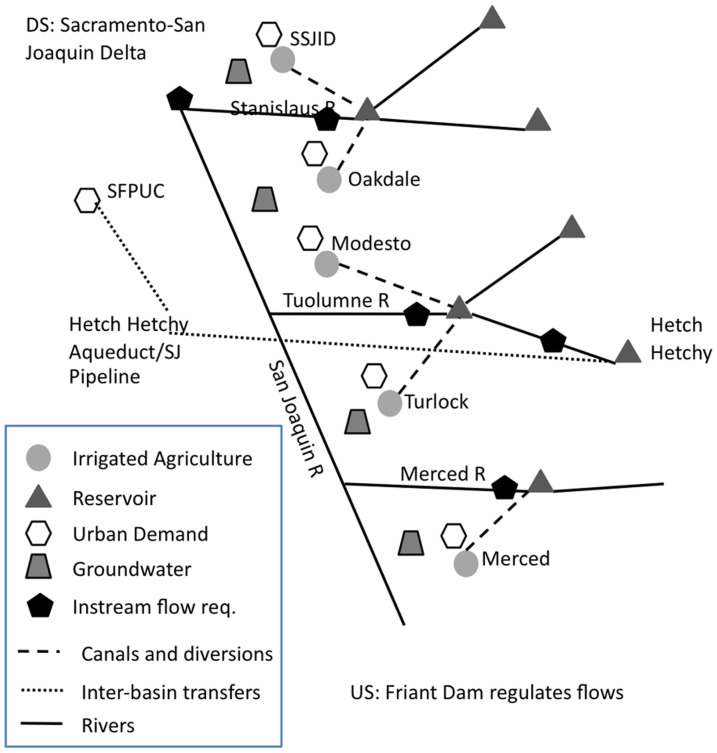
Model schematic. Simplified conceptual schematic of the more detailed model used to represent the river basins.

The WEAP model represents the above-described area with four main streams, 10 reservoir objects, 10 diversions, 4 groundwater basins, 18 distinct agricultural catchments, 88 catchments in the upper watersheds, 15 demands sites, and 19 streamflow requirements. Of these, we focus in this article on those most relevant for the purpose of investigating surface water supplies to agricultural areas, as shown in the highly simplified schematic in Figure 2.

Calibration of this model was guided by the primary study goal of assessment of water supply reliability. The primary calibration point for hydrology was unimpaired streamflow to each major reservoir, as this is the primary hydrologic influence on water reliability. We used historical data for streamflow and reservoir levels from US Geological Survey, California Data Exchange Center, and district sources, as cited in Table S3 in File S1. We also referenced other data sources including sub-catchment streamflow gages and snow surveys for general consistency, but they were not the focus of detailed calibration.

Calibration of unimpaired streamflow, crop demands, and system operation were performed sequentially based on historical data from water years (WY, October–September) 1981–1999. Calibration was performed iteratively, comparing model output with historical data for each watershed. Calibration was aided by Latin Hypercube sampling across parameter space, using Computer Aided Reasoning System software [55]. Further details on calibration and model representation follow.

#### Unimpaired surface water hydrology

**Figure 3:**
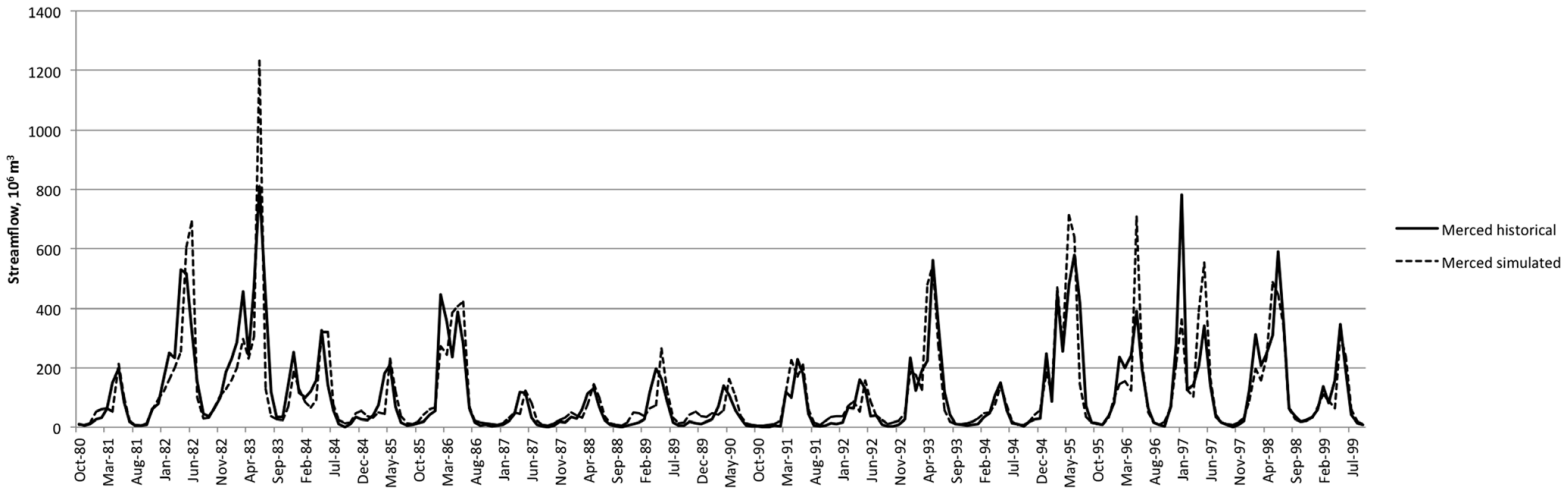
Unimpaired Tuolumne River streamflow. Simulated (dotted) and historical (solid) calculated Full Natural Flows (CDEC gage TLG).

**Figure 4:**
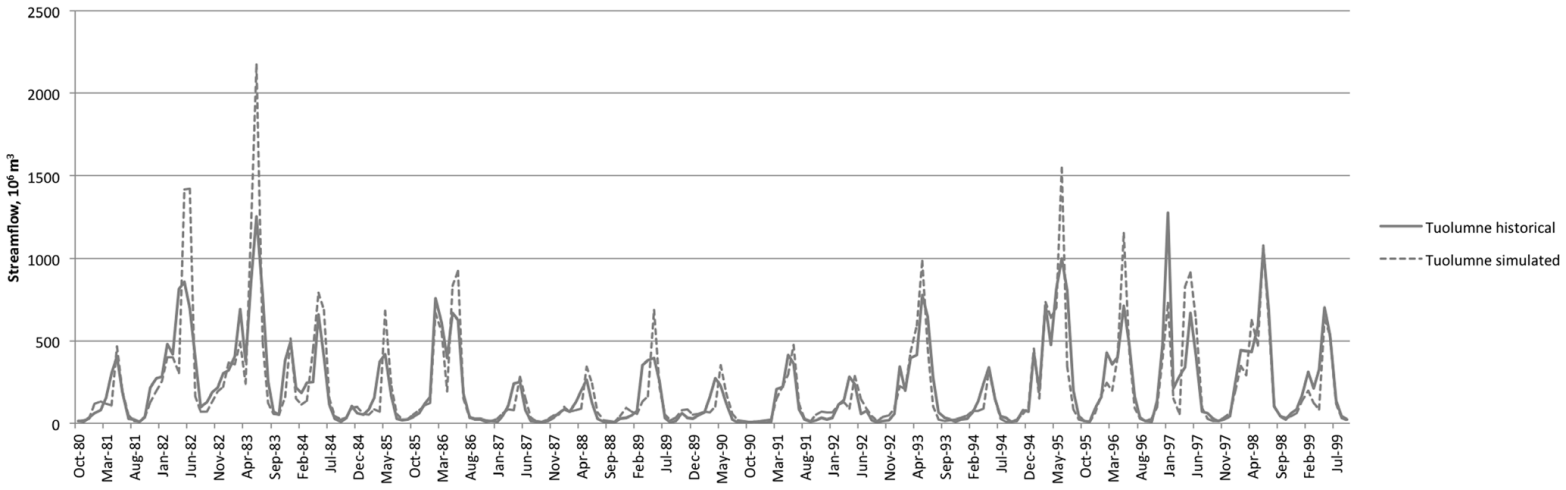
Merced River streamflow (CDEC gage MRC).

**Figure 5:**
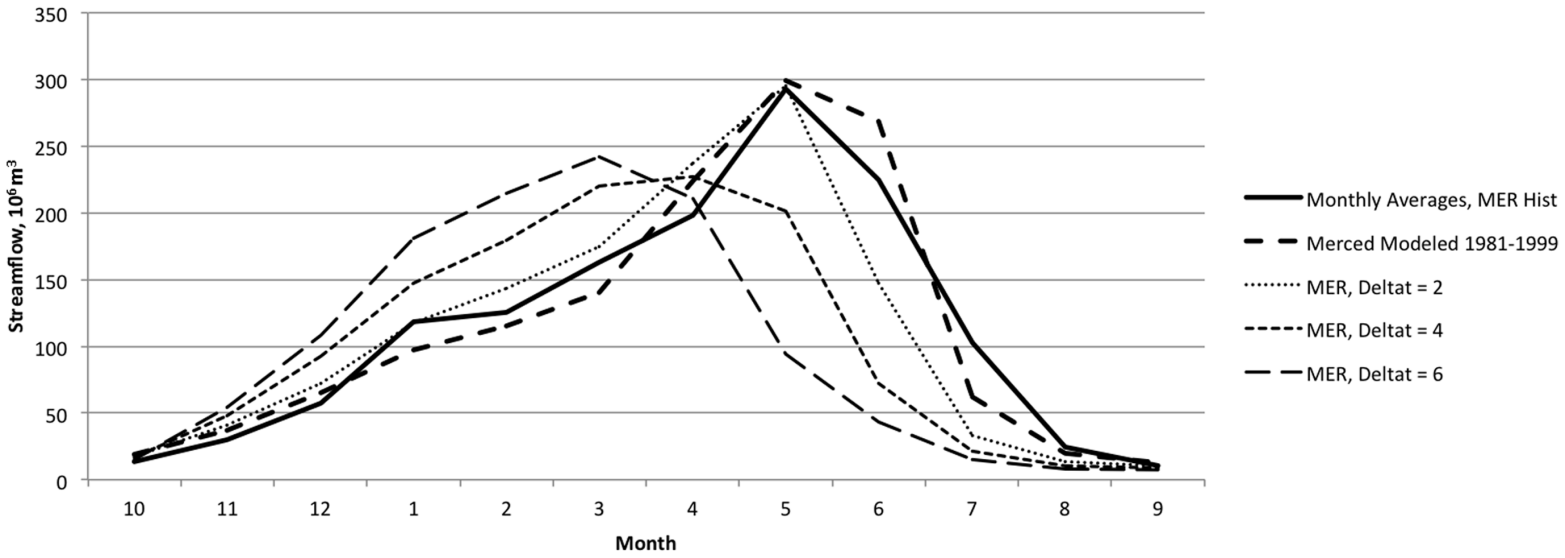
Simulated and historical average monthly unimpaired streamflow. Results (10^6^ m^3^) for the Tuolumne River, as calibrated and in response to temperature increases of 2°C, 4°C, and 6°C.

**Figure 6:**
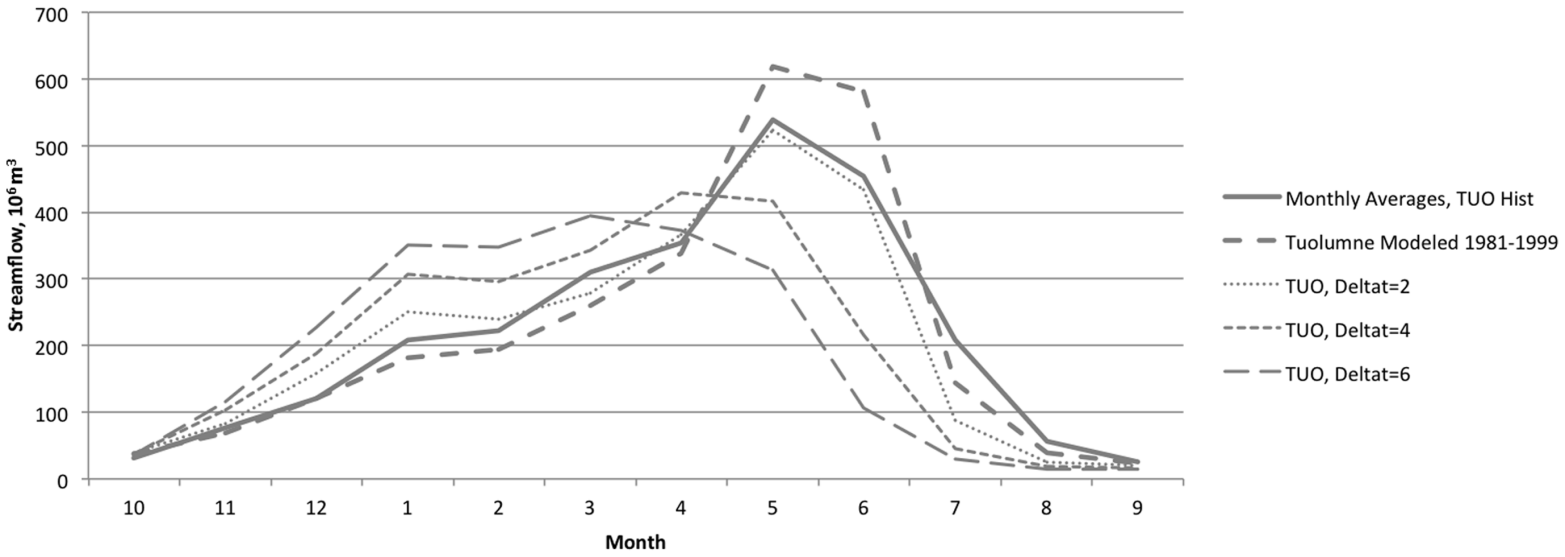
Simulated and historical average monthly unimpaired streamflow. Results (10^6^ m^3^) for the Merced River, as calibrated and in response to temperature increases of 2°C, 4°C, and 6°C.

Figure 3 and Figure 4 depict the model's representation of historical hydrology, comparing WEAP outputs at the pour points representing the large dams at the base of each upper watershed with DWR reconstructed full natural flows. Figure 5 and Figure 6 show average monthly results over the same time period. The model captures historical annual and seasonal variation of flow patterns reasonably well (Table 3). Bias (−2.5% and −0.1%) and goodness of fit (Root Mean Squared Error (RMSE) 67% and 75%) statistics are in the range reported for previous modeling efforts in the region using the Variable Infiltration Capacity model (VIC) [43] and WEAP [20]. Nash-Sutcliffe efficiency index [56] suggests reasonable predictive power for the model.

**Table 3:** Goodness-of-fit statistics for unimpaired hydrology representation for the period from WY 1981–1999.

	TUO	MER
Nash-Sutcliffe	0.67	0.66
Bias (%)	0.1	−0.1
RMSE (%)	66	75

As in previous efforts to model mountainous, snow-driven hydrology with WEAP [21], [57], key parameters adjusted during calibration of streamflow in the upper watersheds include those influencing soil water flux, snowmelt and freezing, along with others as listed in Table 4. These ranges are similar to previously published efforts with WEAP. We used a version of WEAP [11] with an updated value for the latent heat of fusion in the snowmelt algorithm. This enables the use of more physically realistic parameters for melting point than previously published WEAP models.

**Table 4:** Ranges of hydrologic parameters used in the WEAP model.

Parameter	Range
Deep Soil Water Capacity (mm)	732–1339
Root Zone Soil Water Capacity (mm)	288–527
Root Zone Soil Water Capacity (mm)	250
Deep Water Capacity (mm)	200
Runoff Resistance Factors (land-cover dependent)	4–20
Root Zone Conductivity, Deep Soils (mm/month)	43
Root Zone Conductivity, Shallow Soils (mm/month)	331
Deep Conductivity (mm/month)	129–193
Melting threshold (°C)	1
Freezing threshold (°C)	0
Radiation factor	3.5–5.5
Albedo, new snow	0.7
Albedo, old snow	0.03

#### Agricultural demands

Agricultural demands are modeled as a function of climate and crop type using the Penman-Monteith equation and empirically derived crop coefficients [20]. Demand for irrigation water is modeled as a function of reference evapotranspiration (ETo) and non-dimensional crop coefficients (*Kc*) for a given crop. Values for *Kc* were estimated for each crop, tuned from initial estimates for the region found in Bulletin 113-3 [58]. Since observed data in Bulletin 113-3 were produced for the purpose of estimating irrigation requirements rather than modeling year-round hydrology, these data ignore winter-time evaporation, with zero values during non-irrigated months. Missing wintertime *Kc* values were estimated as 0.5. The model simulates irrigation patterns using soil moisture as a trigger for irrigation demands [10]. Flood-irrigated rice uses a separate function to simulate ponding and flushing.

Demands were compared to a metric of Total Applied Water Demand (TAWD), based on estimates for representative irrigation districts in the region [59], where
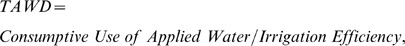
(1)with initial figures taken from Bulletin 113-3 and data from Merced Irrigation District [37]. The above calculations collectively define the amount of water demand at the crop. A key unknown in this region is values for conveyance losses to seepage and evaporation. As in other studies in the region [48], we represent this using a loss factor calibrated to close the water balance, and reflect the total demand for water at the diversion point in each time step. Thus, surface water diversions are modeled as a function of demands for water application, alternative sources of water supply such as groundwater, conveyance capacity, conveyance losses, institutional constraints such as water rights, and reservoir operations [20].

#### Managed water system

Surface water deliveries at the ID or sub-District level are measured at points of diversion from the rivers into the canals that convey water to irrigated and urban demand centers. Modeled deliveries are a function of demands, priorities, preferences, reservoir operations, and available water in a given time step, as described above.

Reservoir operations mediate deliveries to satisfy agricultural water demands for surface water, and in particular to mediate inter-annual variability by reserving water in wet years for use in dry years. The large, terminal reservoirs on these streams also serve flood control functions. Modeling the balance between the two functions was accomplished by adjusting the parameters described in Section 0 above. The top of the buffer zone is defined as a coefficient *b* times the total available storage (conservation level), or as the inactive storage, whichever is greater, with *b* varied during calibration.

An institutional shift took place during the calibration period that influenced operation parameters. All of the reservoirs in this model produce hydropower, and in most cases much of the resulting electricity is sold outside the service areas with revenues benefiting the organizations that also supply water for agricultural purposes. During and after the dry period from 1987–1992, “water first” policies were enacted with the goal of ensuring that system operation hews to its nominal top priority of delivering reliable water supply for constituents. In order to represent this shift to a water first policy, reservoir operations parameters on the Merced and Tuolumne were adjusted before and after 1991. Better fits to historical data were obtained with marginally stricter operating parameters (e.g., higher levels for the buffer zone and/or lower values for the buffer parameter), in keeping with expectations that operational policies would result in greater tendency to store water.

**Figure 7:**
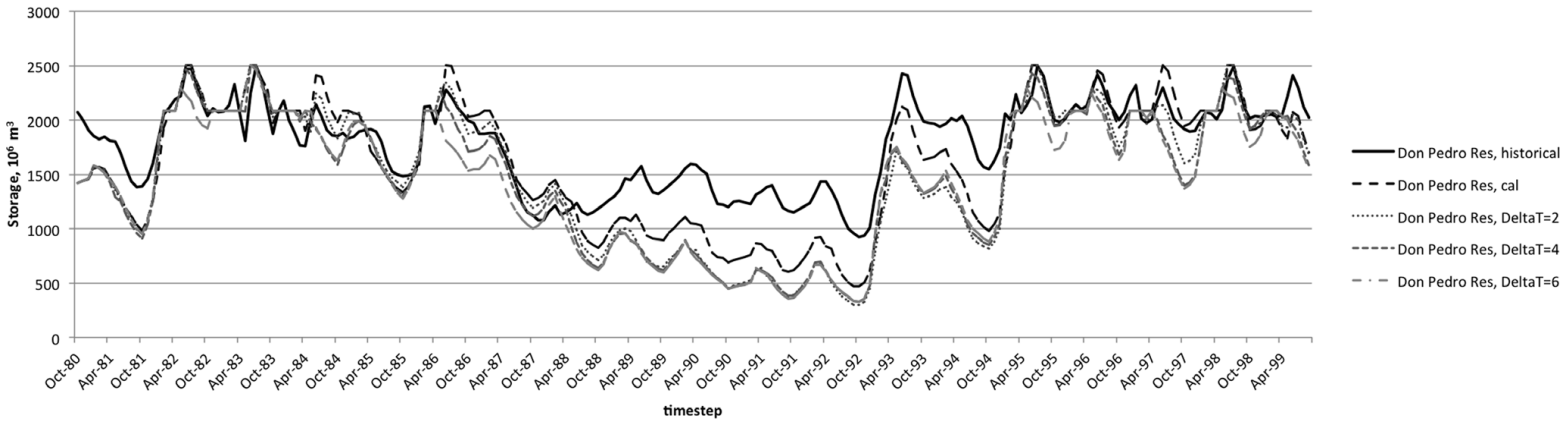
Modeled reservoir levels at Don Pedro terminal reservoir. Results as calibrated to historical observations and modeled with perturbed temperatures of 2°C, 4°C, and 6°C.

The model represents the average annual diversions with reasonable goodness of fit (RMSE ranging from 46–58%, Table 5). While the model exhibits some error in reproducing individual events, it does capture the overall patterns for diversions over the calibration time period, including the shift to lower diversions during the string of critical water years during the drought from 1987–1992 (Table 6). The model captures seasonal and interannual variability in water levels at the terminal reservoirs, with some under-estimation of levels Don Pedro Reservoir (Figure 7, Table 7).

**Table 5:** Goodness of fit statistics for diversions in major canals.

Watershed	Node	Result	RMSE (%)	Nash-Sutcliffe Efficiency Index	Bias (%)
TUO	Modesto Cn	Diversions	46	0.65	4.3
TUO	Turlock Cn	Diversions	46	0.68	2.3
MER	Merced ID N Cn	Diversions	52	0.69	−18.8
MER	Merced ID Main Cn	Diversions	58	0.66	3.0

**Table 6:** Annual historical and modeled diversions to irrigation districts, WY 1981–2000 (10^6^ m^3^).

Basin	Node	Historical average diversions		Modeled average diversions		Model bias (%)	
		1981–1999	1987–1992	1981–1999	1987–1992	1981–1999	1987–1992
TUO	Modesto Canal	379	318	380	312	0	−2
TUO	Turlock Canal	720	581	712	611	−1	5
MER	Merced ID North Canal	26	25	26	20	−2	−23
MER	Merced ID Main Canal	603	482	607	466	1	−3

**Table 7:** Goodness of fit statistics for reservoir inflows, storage, and releases.

Watershed	Node	Result	RMSE (%)	Nash-Sutcliffe Efficiency Index	Bias (%)
TUO	CE	Inflows	101	0.27	−22.5
TUO	CE	Storage	41	−0.27	−0.7
TUO	CE	Releases	103	−0.54	−21.2
TUO	HH	Inflows	84	0.62	−15.0
TUO	HH	Storage	32	0.16	12.2
TUO	HH	Releases	245	−0.17	59.6
TUO	DPR	Inflows	67	0.63	2.5
TUO	DPR	Storage	18	0.37	−8.9
TUO	DPR	Releases	76	0.29	2.0
MER	NE	Inflows	75	0.66	−0.1
MER	NE	Storage	15	0.91	1.0
MER	NE	Releases	76	0.32	0.6

#### Temperature warming scenarios

Climate change assessments can choose from a range of methods for representing modeled sensitivity to future climate change along a continuum of resource intensity [54]. For the present study, we choose to apply a sensitivity analysis to temperature warming. A temperature sensitivity analysis is useful as a first order representation of model behavior, and has been applied in many similar studies in this region and elsewhere. It also serves as a prelude to more detailed analysis in future work. In California, however, there is a reasonable *a priori* expectation that such studies will be meaningful in the face of anthropogenic climate change. First, while climate projections are consistent in their projection of temperature warming for the region, their representation of future precipitation regimes is less so [60], and thus focusing on temperature alone makes sense. Second, since the first studies of mountain hydrology and climate warming in the region, a consistent projection of sensitivity of streamflow to warming has been made in many studies [3], suggesting the usefulness of studying temperature impacts on warming.

We generate a sensitivity analysis using warming scenarios of 2°C, 4°C, and 6°C to bracket expected changes in temperature. As described in Cayan et al. [61] for the Sacramento region, in all projected climate scenarios California retains its Mediterranean temperature and precipitation patterns, with cool wet winters and warm dry summers. Temperature increases between 1°C to 3°C by mid-century, and 2°C to 5°C by end of century, with greater increases in the summer months than in winter. High variability in precipitation evident in the historical and paleoclimate records for the region is also visible in projected climate futures, and the majority of models also show drying trends relative to historical precipitation under each emissions scenario, but we leave exploration of changes in variability to future work.

## Results and Discussion

This section describes the response of modeled hydrology and water operations to the temperature warming scenarios of 2°C, 4°C, and 6°C.

### Hydrology

Under climate warming scenarios, the hydrology of the upper watersheds responds with a shift in timing and magnitude of seasonal flows. As in previous modeling efforts [3], [21], response to simulated warming includes earlier timing of snowmelt and a resulting shift of peak flows earlier in the water year (Figure 5 and Figure 6). Table 8 illustrates this sensitivity through the shift in timing of the center of mass of streamflow. The present model is somewhat less sensitive to temperature by this measure than other efforts [21], and thus may have a relatively muted climate response when compared to other such studies. In addition to the effects on timing, increased temperature decreases magnitude of streamflow (Table 9) through its effect on increased evapotranspiration above the terminal reservoirs (data not shown).

**Table 8:** Simulated shift in hydrograph center of mass (COM).

			Simulated shift in COM, months (days)		
Watershed	Historical COM (1981–1999)	Simulated COM (1981–99)	2°C	4°C	6°C
**TUO**	June 1	June 5	0.17 (5)	0.42 (13)	0.51 (16)
**MER**	May 28	June 1	0.30 (9)	0.51 (15)	0.68 (21)

Shifts earlier in the water year, with uniform temperature increase applied to historical climate inputs over the reference period from 1981–1999.

**Table 9:** Mean annual unimpaired streamflow and its sensitivity to modeled temperature warming (10^6^ m^3^).

Watershed	Historical (1981–1999)	Simulated (1981–99)	Simulated (2°C)	Simulated (4°C)	Simulated (6°C)
TUO	2,608	2,609	2,506 (−3.9%)	2,418 (−7.3%)	2,323 (−11%)
MER	1,363	1,361	1,303 (−4.3%)	1,245 (−8.5%)	1,195 (−12.1%)

### Water supply reliability

For the agricultural districts modeled here, surface supply reliability is reduced under uniform temperature warming of 2°C, 4°C, and 6°C. This is a function of changes in agricultural water demands and hydrology mediated by the modeled behavior of storage, conveyance and irrigation systems.

Reliability metrics can assign a binary metric for each iteration, where a given time point is determined either to a failure or success state based on a threshold condition, and reliability is a probabilistic measure of rate of success [62], [63]. We use the quantity-based reliability measures suggested by Dracup et al. [64], which measure degree of failure based on the amount of shortfall below the threshold:
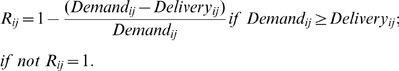
(2)This metric is collapsed into an a overall reliability measure
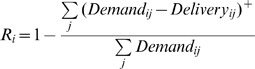
(3)where *i* represents a given demand point or group of demand points, and *j* represents timesteps. The ^+^ indicates that negative values are replaced with zero values so as not to bias the analysis in the case of overdelivery at a given time step.

As described above, the model includes a historically based preference for some groundwater supply for each agricultural demand node. We normalized the reliability calculations to exclude this groundwater supply such that groundwater use forced by historical preference does not reduce reliability statistics for surface water. This may skew estimates of overall reliability, as we do not separate preference for groundwater by users within an irrigation district from use of groundwater in response to shortages.

Table 10 shows the trajectory of modeled supply reliability at each of the major irrigation districts in the basins under the temperature warming scenarios. Reliability decreases marginally with increasing temperature in each case, even in absence of changes in precipitation patterns. These changes are driven in part by the changes in streamflow described above. In addition, modeled demands increase for 2°C (1.4–2.0%), 4°C (2.8–3.9%), and 6°C (4.2–5.8%). The values for this sensitivity analysis result from representation of agricultural demands via the Penman-Montieth equation, which is sensitive to temperature. These results do not take into account the potential for other physical (e.g., plant physiological response to increases in CO_2_ concentrations) or behavioral changes (e.g., changes in cropping patterns or irrigation technology), and could be refined in future efforts.

**Table 10:** Modeled surface supply reliability decreases with increasing temperature, based on the reliability metric described in the text.

	ΔT			
	0°C	2°C	4°C	6°C
Modesto ID	0.84	0.82	0.79	0.75
Turlock ID	0.86	0.85	0.82	0.79
Merced ID	0.90	0.86	0.81	0.75

**Figure 8:**
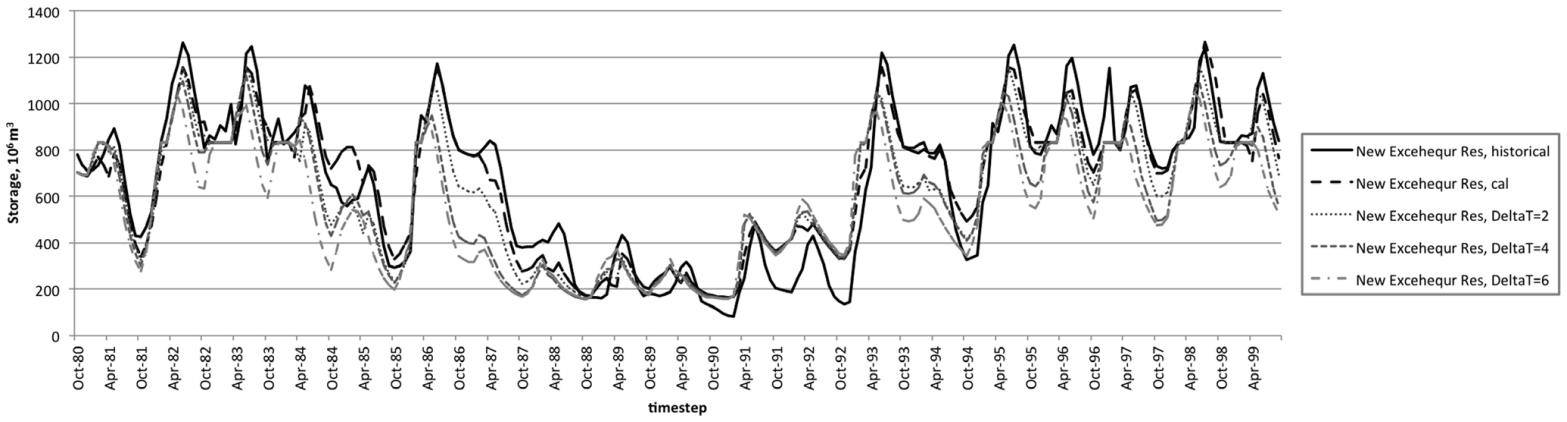
Modeled reservoir levels at New Exchequer terminal reservoir. Results as calibrated to historical observations and modeled with perturbed temperatures of 2°C, 4°C, and 6°C.

The influence of temperature-driven changes in hydrology and agricultural water demands are apparent in modeled reservoir levels (Figure 7 and Figure 8). In each of the terminal reservoirs, increasing temperature inputs results in lower reservoir levels on average over the calibration period (Table 11). The effect is different in each basin, however. In New Exchequer, the storage decreases are minimal during drought years, while the reverse is true in Don Pedro. This may be a result of the operations behavior during this time period. For example, New Exchequer's smaller buffer zone allows it to be drawn down low initially during the drought period, leaving less potential for meeting demands later in the irrigation season, while Don Pedro has somewhat more gradual initial drawdown and more room for change. Note that the underestimation of Don Pedro Reservoir levels in calibrated results suggests that reliability impacts may be overestimated in the modeled scenarios, although this is likely not a major issue in light of the size of the reservoirs relative to demands. While shifts in streamflow timing and magnitude result from the modeled temperature changes, the large storage volume in these reservoirs allows them to meet demands with a fairly small loss of reliability. Thus, reliability is somewhat, but not dramatically, sensitive to the modeled temperature changes. Since temperature change is only one of the many relevant climate variables projected to affect water resources [9], future work in this system will examine system sensitivity to models forced by General Circulation Models that incorporate changes in variability of temperature and precipitation in more nuanced ways.

**Table 11:** Mean reservoir levels and response to temperature increases.

ΔT	New Exchequer Res	Don Pedro Res
**Historical**	666	1784
**Modeled**	671	1626
**2°C**	625 (−6.8%)	1520 (−6.6%)
**4°C**	590 (−12.1%)	1483 (−8.8%)
**6°C**	550 (−17.9%)	1448 (−10.9%)

Table shows average reservoir storage volumes (10^6^ m^3^) at the terminal reservoir for each stream. Percent decrease from calibrated reservoir volume is shown in parentheses.

Water resources infrastructure and policies are designed in part to reduce the risks inherent to climatically driven, variable water resources systems. In short, they are built for adaptation to climate variability. A crucial question for water resources managers is how water resources systems built with the purpose of reducing the impacts of climate variability such as droughts and floods will help these water systems reduce the risks from changing climatic conditions.

While the conceptual question is a general one, the answers will necessarily be place-based and situation-specific. Extensive work has been done investigating and characterizing the potential changes that may result in California's snowmelt-driven hydrology under future climatic non-stationary. The present study joins others in developing a case study with integrated modeling techniques capable of analyzing the impacts of such streamflow changes on the combination of physically-driven streamflow with built infrastructure and its operation.

The results presented above suggest that increased temperatures within the range of what is expected over the course of the 21^st^ century could affect the chief nominal goal of the modeled water resources system, namely the provision of reliable water supply for use in agriculture. Whether these representations are inherent characteristics of institutional policies or artifacts of the model structure and parameterization, hydrologic change introduced by increasing temperatures results in decreases to modeled reliability of water supply.

In sum, modeled impacts of warming scenarios from 2–6°C affect streamflow magnitude (decreases from 4–12%, Table 9), timing (shifts from 5–21 days earlier, Table 8), while increasing agricultural demands by 1.4–5.8%. The net effect of these changes is that modeled surface water supply reliability decreases in each district, but less than might be expected were the reliability response a simple summation of supply and demand changes (Table 10). The substantial reservoirs providing storage intended to buffer the effects of climate variability serve to reduce, but not eliminate, the hydrologic impacts of climate change in the same way as they offset short-term hydrologic droughts.

While the built system in this model contributes to attenuation of the signal of climate change, it does not do so completely. Impacts are still apparent in the form of reduced surface water supplies in dry years. While the reliability of the system remains high under temperature warming, such a shift may be enough to cause concern among water managers.

### Model appropriateness and limitations

The strengths and limitations of a related WEAP application have been discussed by Yates et al. [20], including static land use, the origin of the climate inputs, and some simplifying assumptions in representing agriculture and reservoir operations. The limitations discussed by Yates et al. [20] apply generally to the present work as well, which is based on similar methods. We expand on some of these observations here, while noting that given a conceptual continuum from oversimplified and lacking nuance to a 1∶1 scale map that is highly representative but cumbersome and possibly intractable, the present exercise attempts to strike a suitable balance for the intended purpose of long-range sensitivity analysis to major system-scale stressors such as climate change.

To simulate changing deliveries under varying water availability, reservoir operations rules limit surface supplies when reservoir storage falls below a specified level. When surface water use is curtailed, for example when low reservoir levels trigger delivery limitations, simulating concern for carryover storage for the next year, the remaining demands are supplied through groundwater pumping. Currently, there is no constraint on groundwater use in the model. Incorporating better data on district-wide pumping capacity, or assumptions of future pumping capacity, could better describe groundwater pumping limits.

Reproducing fine-scale weather patterns is notoriously difficult, especially in mountainous terrain. With data at 1/8° resolution, nuance can be expected to be lost, as described above in the climate inputs section. While the model performs well overall in reproducing historical streamflow, in some areas bias might result from input data.

Spatial and temporal scales are relatively coarse. We chose to implement this model on a monthly time step because of the management relevance for long term planning applications, because some data are only available at monthly time steps, and because computational limitations would make long-range projections intractable using smaller timesteps. The monthly time step limits the resolution for individual events. Thus, analysis of flood risk, sub-monthly instream-flow requirements such as pulse flows, and other applications requiring finer-scale hydrology must be left to other platforms. Hydrology is modeled in WEAP using a quasi-physical lumped parameter approach, whereby land classes within each catchment object are combined and assigned common hydrologic responses. A more detailed approach might, for example, assign land classes and hydrologic response parameters to each segment of a grid covering the model domain, and route water between points on the grid [65]. However, others have argued that more detail inherently comes with the disadvantage of increasing uncertainty intrinsic to larger numbers of estimated parameters [66], [67].

Water operations in WEAP enable the model to represent satisfaction of competing current and future demands for water. Operations are defined through a combination of logical constraints (e.g. minimum instream flow requirements) and more general characteristics (e.g. reservoir operations parameters). The latter represents what in reality is a complex set of decisions that even in historical representation includes factors outside the model domain such as economics, long-range weather forecasts, political decisions, changing legal constraints, and so forth. One can think of the buffer concept as a way to represent the general degree of ‘conservatism’ in operations decisions. It has the advantage of flexibility, and the disadvantage that details of operational decisions and changes in reservoir operations logic can only be represented in a broad-brush sense. For long range planning scenarios, this approach is a valid one given the tremendous uncertainty that exists in the details of future policy decisions. In addition, a strength of this approach is the flexibility with which general changes in policy such as adaptive changes in flood rules or reservoir tolerances could be modeled as part of future efforts, suggesting that the tool described here provides the basis for meeting a recognized need for future climate impact and adaptation studies [3].

Future work could implement changes in agricultural water use efficiency [68], response of cropping patterns to drought [23], [69], and change in crops to favor products with higher economic value [70]. Also, the modeling framework is designed for future implementation of more detailed scenario analyses driven by downscaled GCM data [50], although such analysis is beyond the scope of the current paper. We also leave model structure uncertainty and hydrology parameter uncertainty [71] for later work.

## Conclusion

In this paper, we have presented a tool for studying climate impacts and adaptation in California water resources. The model represents the hydrology and water operations of the Tuolumne and Merced River Basins in California's San Joaquin Valley. Although climate change is a global environmental catastrophe, the impacts of greatest interest to humans will be its local manifestations, and particularly those manifestations that result in direct consequences to the systems that directly support natural and man-made processes vital to life on earth. Translation of climate impacts most relevant to human decision-making will involve case studies at local levels. Thus, a contribution of this paper lies in its value as a case study for such translation not only in terms of scale, but of topic, from proxies for climate warming to reservoir operations and agricultural water supply reliability.

The WEAP model described in this paper represents annual and seasonal variability in hydrology and water operations, and enables the development of analysis of future water conditions using projections of climate change, land use change, and population growth. The results presented here illustrate system attenuation of the climate change signal: as impacts move to higher order impacts, flexibility in the system buffers the response.

Whether reliability changes of the magnitude estimated here are viewed as significant will depend on the perspective of water managers and water users. For example, water managers often tend to be averse to downside risk [72], and such risk aversion in effect amplifies the felt impacts of supply shortfalls. Formal approaches to answering these questions could include addressing in more detail formal elicitation of the risk preferences and value functions of water users and water managers, and detailing the ultimate impacts felt by users such as crop failure or additional costs incurred for pumping. However, reliability estimates such as those described here may inform anticipatory adaptation actions such as investment in increased water use efficiency measures, the use of crop insurance, and other measures [9].

Nonetheless, impacts on reliability are visible with temperature warming, suggesting value in future work that will also move along the continuum of climate analyses described by Wilby et al. [54]. Such efforts will incorporate downscaled GCM models, and enable us to reflect changes in variability of temperature and precipitation for more detailed climate impacts analysis.

## Supporting Information

File S1 Supporting methods and tables, including logic for operations, forecasting, and flow constraints such as in-stream flow requirements and hydropower.
(DOCX)
