# Supplementary material for: Potential Impacts of Climate Warming on Water Supply Reliability in the Tuolumne and Merced River Basins, California
Source: PLoS One. 2014 Jan 20;9(1):e84946. doi: 10.1371/journal.pone.0084946 (PMC3896353; doi:10.1371/journal.pone.0084946)
Supplement: File S1 — Supporting methods and tables, including logic for operations, forecasting, and flow constraints such as in-stream flow requirements and hydropower. (DOCX) [file pone.0084946.s001.docx]

**Supporting Information for:**

**Potential impacts of climate warming on water supply reliability in the Tuolumne and Merced River Basins, California**

Michael Kiparsky, Brian Joyce, David Purkey, Charles Young

# Supporting information

The following supporting information details logic for operations, forecasting, and flow constraints such as in-stream flow requirements and hydropower.

## Stanislaus River in-stream flows

The New Melones Reservoir is operated for four purposes, fishery, water quality, Bay-Delta flow, and water supply, as formalized in the New Melones Interim Plan of Operations [[1](#_ENREF_1)].

### Forecasting the Stanislaus Year Type Index

In practice, flow requirements for the year are based on New Melones storage at the end of February, plus forecasted inflows to New Melones for March-September. We operationalized the forecast data by regressing March 1 modeled snowpack^[[1]](#footnote-1)^ over the period from 1950-1999 against modeled March-September unimpaired flows in the Stanislaus River (R^2^ = 0.72) to approximate a snowpack-based forecast.

We used this linear model as a heuristic representation of the forecast aspect of these indices, while acknowledging that the actual California Department of Water Resources (DWR) forecast is based on other factors than snow surveys [[2](#_ENREF_2)]. It is also important to note that this method (and the forecasting described in Section 1.2.1 and Section 1.3.1) assumes climate stationarity [[3](#_ENREF_3)], which will not actually apply to future climate scenarios. Future work could address this by updating forecasting logic as the simulated hydrologic ‘record’ lengthens over the course of each climate change scenario.

The Water Evaluation and Planning Model (WEAP) can return calculated values for a previous (but not current) timestep for use in calculations. Thus, there is a one-month lag in calculations using this forecast (e.g. April 1997 will calculate In-stream Flow (ISF) requirements based on the previous year’s forecast, and May 1997 will use the current year forecast).

The Stanislaus River index is used to determine minimum release schedules for each of the four purposes of the New Melones Reservoir. Priorities for each ISF requirement are defined such that flow requirements are satisfied in the following order in each time step: 1) fisheries; 2) Proxy for D-1641 Bay-Delta flow requirements at Vernalis; 3) water supply. When water is not available for all of these purposes, the lower priority uses will be shorted first. As described below in Section 1.4, required Bay-Delta and Vernalis Adaptive Management Program (VAMP) flows are included within flow constraints specified through State Water Resources Control Board (SWRCB) and DWR modeling.

### Stanislaus River fishery flows

Fishery flows are based on flow requirements of the 1987 Reclamation, Department of Fish and Game (DFG) Agreement and prescriptive use of Central Valley Project Improvement Act (CVPIA) 3406(b)(2) [[1](#_ENREF_1)]. In practice, requirements are linearly interpolated between values for each year type. Here, we use the value for each year type as defined in Table S5 and S6.

### Stanislaus River D-1422 flows

SWRCB Decision 1422 (D-1641) [[4](#_ENREF_4)] requires water to be released from New Melones reservoir to keep dissolved oxygen concentration above 7 mg/l at Ripon. The current WEAP model does not estimate water quality parameters. Instead, we use fixed releases at low-flow times of the year as a surrogate for these flows (Table S7), as represented in the CALSIM model [[1](#_ENREF_1)]. The United State Bureau of Reclamation (USBR) bears substantial responsibility for meeting water quality and flow requirements at Vernalis through releases from New Melones Reservoir. See Section 1.4 on page 4 for details.

## Tuolumne River in-stream flows

### 60-20-20 Index forecasting

In-stream flow requirements in the Tuolumne River below Don Pedro Dam vary based on DWR’s San Joaquin River (SJR) 60-20-20 index [[5, p. 24](#_ENREF_5)]. The 60-20-20 index is computed for each year by using actual and forecasted values in the following equations:

INDEX = 0.6 * X + 0.2 * Y + 0.2 * Z, where Equation 1

X = Current year’s April – July San Joaquin Valley unimpaired runoff (MAF)^[[2]](#footnote-2)^

Y = Current October – March San Joaquin Valley unimpaired runoff (MAF)

Z = Previous year’s index (up to a maximum of 4.5 MAF)

The geographical scope of the WEAP model presented here contains three of the four river basins included in this index (inflow to Millerton Lake on the San Joaquin River is not included in the current model). Thus, we developed an analogue by comparing output for the three basins in the WEAP model to historical data for each element of the index, and generating scaling factors for each element.

To generate scaled values for Y (Oct-Mar inflows), we ran the WEAP model from WY 1950-1999 (the period for which historical climate data were available), and regressed modeled full natural flows in the Stanislaus, Tuolumne and Merced Basins against historical full natural flows for the SJR Basin (R^2^ = 0.89), and used the coefficient from this linear model (1.59) as a scaling factor for generating Y from each year’s modeled data.

In practice, X is based on forecasted inflows, which depend substantially on snowpack measurements, among other factors. We used a Geographical Information System (GIS) to select a sample of catchments near DWR’s snow course sensors^[[3]](#footnote-3)^. The model sums snow depths in the sample catchments to make an index of April 1 snow accumulation. We regressed modeled snow accumulation at Mar 1 time steps from 1950-1999 against modeled historical Apr-Jul SJR Basin flows (R^2^ = 0.60), and used the results to scale modeled snowpack into the annual value for X.

SWRCB defines five year types for the SJR 60-20-20 Index (Table S8). As an illustration of the variability in the relationship between index, modeled, and historical values, over the 50 water years from 1950-1999 the index matches the historical actual flow index 31 times (62%) and comes within one step of the historical index 45 times (90%).

### Tuolumne River in-stream flow requirements

Tuolumne River ISF requirements below New Exchequer Reservoir are based on Federal Energy Regulatory Commission (FERC) rules [[6](#_ENREF_6)].^[[4]](#footnote-4)^ FERC defines seven year type descriptions with distinct corresponding flow prescriptions (Table S9). These year types differ from the SWRCB/DWR water year types described above (Table S8). In practice, FERC year type designations to be updated as the hydrologic record increases in length, and exact flow amounts are defined based on the exact 60-20-20 index value using interpolation between the values in Table S9. The current WEAP model uses the values in Table S9 directly based on threshold values for the index, and does not update for changes in distribution of index values over time, generally resulting in an underestimate of required flows.

## Merced River in-stream flows

### Merced River year type forecasting

In practice, two year types are defined for the Merced River based on forecasting. A normal year as defined by the FERC license for the Merced River occurs when forecasted April through July inflow to Lake McClure is equal to or greater than 555 mcm (450 TAF), as published in DWR May 1 Bulletin 120 [[7](#_ENREF_7)]. A dry year as defined by FERC license is when forecasted April through July inflow to Lake McClure is less than 555 mcm (450 TAF), as published in DWR May 1 Bulletin 120.

To simulate forecasting logic, we regressed an index for May 1 Merced River basin modeled snowpack against modeled June-July inflow to Lake McClure for WY 1950-1999 (R^2^= 0.97). We then used this linear relationship to model forecasted flows, add them to modeled April-May flows, and determine year type based on the threshold described above. The modeled forecast from WY 1950-1999 matches the actual DWR year type forecast 88% of the time, compared to a historical accuracy of the DWR forecast of 94% over the same time period (historical forecast data obtained from Steve Nemeth, DWR, pers. comm.).

### Merced River in-stream flow requirements

On the Merced River, in-stream flows are required for the Cowell Agreement Entitlement between Merced Irrigation District (ID) and senior downstream riparian users, Federal Energy Regulatory Commission (FERC) requirements, and the Davis-Grunsky contract between the State of California and Merced ID [[8](#_ENREF_8)].

To satisfy instream flows, “Merced I.D. operates to a target flow below Crocker-Huffman diversion dam equal to the Cowell Agreement adjudicated entitlement plus the FERC/Davis-Grunsky flow requirement. The flow below Crocker-Huffman Diversion Dam must be equal the greater of the Davis-Grunsky and FERC flows plus the Cowell Agreement Entitlement” [[8](#_ENREF_8)]. Flow requirements are shown in Table S10.

## Delta water quality requirements

### Flow requirements at Vernalis

Vernalis marks the southern-most boundary of the legal Bay-Delta, and as such serves as a control point for water quality regulations for water flowing north into the Delta from the San Juoquin River. There are two main drivers of flow requirements at Vernalis; D-1641 and the Vernalis Adaptive Management Program (VAMP) [[9](#_ENREF_9)].

### SWRCB D-1641 and VAMP

D-1641 [[4](#_ENREF_4)], issued in 1999, accepts negotiated contributions of water made by other parties (e.g. water rights holders) towards meeting the goals of the 1995 Bay-Delta Water Quality Control Plan [[5](#_ENREF_5)]. The goals of the 1995 Bay Delta Plan are to protect beneficial uses through water quality objectives, specifically by managing salinity intrusion, dissolved oxygen, and flows and diversion in the Delta.

Because of the limited spatial extent of this modeling effort, because water quality modeling is outside the scope of this exercise, and because “factors to be controlled [by D-1641] are primarily related to flows and diversions” by water rights holders [[5, p. 4](#_ENREF_5)], we used data from SWRCB modeling to simulate the contribution of each modeled basin to Vernalis flows. D-1641 requires the flow at Vernalis to be maintained during the February through June period based on the required location of the Delta salinity index X2 and the San Joaquin Basin Index. Endogenizing the Delta salinity trigger is beyond the scope of the present modeling. Instead, we have taken advantage of modeling studies carried out during the Environmental Impact Report process for D-1641. SWRCB and DWR conducted modeling using DWRDSM to determine the potential contributions of each watershed in the Central Valley to meeting potential Delta water quality requirements. We used the results of their modeling [[9](#_ENREF_9),[10](#_ENREF_10)] as a proxy for flows released to meet water quality requirements, as depicted in Table S11. Note that in the Stanislaus River, these requirements were scaled upwards by 30% to better match observed releases.

### Further details

Post-processing and data analysis was accomplished with the R statistical package [[11](#_ENREF_11)].

# Supplemental Tables

| **NLDC Codes** | **Land Use Classification** |
| --- | --- |
| 11,12, 90-99 | Water |
| 21,22,23,23 | Urban |
| 31,32 | Barren |
| 41,42,43, | Tree |
| 52 | Shrub |
| 71 | Grassland |
| 81,82 | Agriculture |

Table S1: Simplified land use/land cover classifications for the upper watersheds, based on National Land Cover Dataset (NLCD) data.

| **WEAP Agricultural Land Use Classifications** |
| --- |
| Grain |
| Rice |
| Cotton |
| SgrBeet |
| Field |
| Alfalfa |
| Pasture |
| Pr_Tom |
| Fr_Tom |
| Truck |
| Orchard |
| Subtrop |
| Vine |
| Fallow |
| Other_Non_irr |

Table S2: Land use/land cover classifications for hydrologic and demand modeling in the Valley floor, based on mapping from DWR spatial land cover surveys to a simplified version of annual land and water use survey classes ([www.landwateruse.water.ca.gov](http://www.landwateruse.water.ca.gov)).

| **Reservoir Object** | **Reservoir Name(s)** | **Total Storage Capacity (TAF)** | **Data Source** |
| --- | --- | --- | --- |
| NE | Lake McClure | 1,032 | Merced ID |
| CE | Cherry Lake  Lake Eleanor | 303 | 11277500, 1127200, 11278400, SFPUC |
| HH | Hetch Hetchy | 360 | 11275500, 11276500, SFPUC |
| CCSFWB | City and County of San Francisco Water Bank in Don Pedro Res | 570 |  |
| DPR | Don Pedro Res | 1460 | 11287500, 11289651, SFPUC |
| PL | Strawberry Lk Lyons Res | 23 | 11297700, 11295900, 11298000, 11297500, 11297000 |
| BDR | Beardsley Lk Donnells Res Relief Res | 180 | 11292600, 11291000, 11292800, 11292901 |
| NSM | New Spicer Meadows Res; McKays Point Res; Utica Res; Union Res; Lake Alpine | Before 1989: 13  After 1989: 195 | 11293770, 11295260, 11293460, 11293350, 11293370, 11295300, 11295240, 11295505 |

Table S3: Physical characteristics of reservoir objects in WEAP model, describing lumping of upper reservoirs for computational efficiency. Data sources for calculation of storage, inflows, and releases refer to USGS gages and California Data Exchange Center. Time series vary in length. Only the major reservoirs are discussed in this article, as the smaller ones have minor effects on the operations of focus.

| **Urban Node** | **Corresponding agricultural area** | **Urban Areas Represented** | **Population estimate (thousands)** | |
| --- | --- | --- | --- | --- |
|  |  |  | 1980 | 2000 |
| Turlock Urban | Turlock ID | Turlock and vicinity | 124 | 163 |
| Merced Urban | MercedID (South) | Merced, Atwater, Livingston and vicinity | 112 | 138 |
| Oakdale Urban | Oakdale ID | Oakdale and vicinity | 28 | 35 |
| Manteca Urban | SSJID | Manteca, Ripon and vicinity | 42 | 67 |
| Stockton Urban | Stockton East WD | Stockton and vicinity | 273 | 311 |
| Modesto Urban | Modesto ID | Modesto and vicinity | 154 | 223 |

Table S4: Description of urban center nodes and population estimates.

| **WEAP fisheries flow schedule** | **A** | **B** | **C** | **D** | **E** | **F** | **G** |
| --- | --- | --- | --- | --- | --- | --- | --- |
| **New Melones Storage plus Inflow threshold (TAF)** | 0 | 1400 | 2269 | 2293 | 2421 | 2767 | 3000 |
| **Fishery Flow Determination** | 0 < index =< 1400 | 1400 < index =< 2269 | 2269 < index =< 2293 | 2293 < index =< 2421 | 2421 < index =< 2767 | 2767 < index =< 3000 | > 3000 |
| **Fisheries allocation (TAF, approx.)** | 0 | 98.4 | 243.3 | 253.8 | 310.3 | 410.2 | 466.8 |

Table S5: Logic for determination of WEAP Stanislaus River minimum fishery flow schedules (cfs). Note that in practice requirements are linearly interpolated between the seven distinct flow schedules, while in the current model we use thresholds to determine which of the flow schedules in Table S6 is instituted. This results in a bias towards smaller fishery flow requirements. Index values refer to the Stanislaus River Index, as described above (TAF).

|  | **Fishery Flow Schedules with Flow Requirements (TAF)** | | | | | | |
| --- | --- | --- | --- | --- | --- | --- | --- |
|  | **A** | **B** | **C** | **D** | **E** | **F** | **G** |
| **October** | 0 | 110 | 200 | 250 | 250 | 350 | 350 |
| **November** | 0 | 200 | 250 | 275 | 300 | 350 | 400 |
| **December** | 0 | 200 | 250 | 275 | 300 | 350 | 400 |
| **January** | 0 | 125 | 250 | 275 | 300 | 350 | 400 |
| **February** | 0 | 125 | 250 | 275 | 300 | 350 | 400 |
| **March** | 0 | 125 | 250 | 275 | 300 | 350 | 400 |
| **April** | 0 | 375 | 900 | 900 | 1200 | 1500 | 1500 |
| **May** | 0 | 375 | 900 | 900 | 1200 | 1500 | 1500 |
| **June** | 0 | 0 | 200 | 200 | 250 | 800 | 1500 |
| **July** | 0 | 0 | 200 | 200 | 250 | 300 | 300 |
| **August** | 0 | 0 | 200 | 200 | 250 | 300 | 300 |
| **September** | 0 | 0 | 200 | 200 | 250 | 300 | 300 |

Table S6: WEAP representation of Stanislaus River fishery flow schedules. April and May monthly flow requirements are approximated by averaging the monthly scheduled flow with the April-May pulse volume to get an average monthly flow rate.

| **Month** | **Surrogate release volume (TAF)** | **ISF (cfs)** |
| --- | --- | --- |
| Jun | 13.2 | 222 |
| Jul | 16.2 | 263 |
| Aug | 16.4 | 267 |
| Sep | 14.3 | 240 |

Table S7: Surrogate release volumes for D-1422 water quality requirements at Ripon on the Stanislaus River (TAF) and corresponding minimum flows in WEAP (cfs). After Table 6-8 in [[1](#_ENREF_1)].

| **Year Type** | **60-20-20 Index Value** |
| --- | --- |
| Wet | 3.8 =< Index |
| Above Normal | 3.1 < Index =< 3.8 |
| Below Normal | 2.5 < Index =< 3.1 |
| Dry | 2.1 < Index =< 2.5 |
| Critical | Index =< 2.1 |

Table S8: SWRCB San Joaquin Valley Water Year Hydrologic Classification. State Water Resources Control Board water year types for the San Joaquin Basin. These year types are used for the designation of Delta flow requirements, among other purposes [[5](#_ENREF_5)].

| **Year Type** | **Unit** | **Critical & below** | **Median Critical** | **Interm. CD** | **Median Dry** | **Interm. D-BN** | **Median Below Normal** | **Interm. BN-AN** |
| --- | --- | --- | --- | --- | --- | --- | --- | --- |
| **60-20-20 Index threshold** | TAF | <1500 | 1500 | 2000 | 2200 | 2400 | 2700 | >3100 |
|  |  |  |  |  |  |  |  |  |
| **Month** |  |  |  |  |  |  |  |  |
| Oct | Cfs | 125 | 125 | 150 | 150 | 180 | 188 | 300 |
| Oct | Attraction pulse (AF) | none | none | none | none | 1676 | 1736 | 5950 |
| Nov | Cfs | 150 | 150 | 150 | 150 | 180 | 175 | 300 |
| Dec | Cfs | 150 | 150 | 150 | 150 | 180 | 175 | 300 |
| Jan | Cfs | 150 | 150 | 150 | 150 | 180 | 175 | 300 |
| Feb | Cfs | 150 | 150 | 150 | 150 | 180 | 175 | 300 |
| Mar | Cfs | 150 | 150 | 150 | 150 | 180 | 175 | 300 |
| Apr | Cfs | 150 | 150 | 150 | 150 | 180 | 175 | 300 |
| Apr | Outmigration pulse (AF) | 5546 | 10046 | 16310 | 18530 | 17960 | 30014 | 44941 |
| May | Cfs | 150 | 150 | 150 | 150 | 180 | 175 | 300 |
| May | Outmigration pulse (AF) | 5546 | 10046 | 16310 | 18530 | 17960 | 30014 | 44941 |
| Jun | Cfs | 50 | 50 | 50 | 75 | 75 | 75 | 250 |
| Jul | Cfs | 50 | 50 | 50 | 75 | 75 | 75 | 250 |
| Aug | Cfs | 50 | 50 | 50 | 75 | 75 | 75 | 250 |
| Sep | Cfs | 50 | 50 | 50 | 75 | 75 | 75 | 250 |
| **Volume** | **AF** | **94000** | **103000** | **117016** | **127507** | **142502** | **165002** | **300923** |

Table S9: WEAP implementation of FERC instream flow requirements at LaGrange on the Tuolumne River. October attraction pulse flows and April and May outmigration pulse flows for salmon are approximated as additional required flow rates divided evenly between April and May.

| **Month** | **Davis Grunsky Crocker-Huffman Dam to Shaffer Bridge**  **(cfs)** | **Normal Year (cfs)** | **Dry Year (cfs)** | **Cowell Agreement Entitlement (cfs)** | **Total Normal Year (cfs)** | **Total Dry Year (cfs)** |
| --- | --- | --- | --- | --- | --- | --- |
| Oct | 0 | 50 | 37.5 | 50 | 100 | 87.5 |
| Nov | 180-220 | 100 | 75 | 50 | 250 | 250 |
| Dec | 180-220 | 100 | 75 | 50 | 250 | 250 |
| Jan | 180-220 | 75 | 60 | 50 | 250 | 250 |
| Feb | 180-220 | 75 | 60 | 50 | 250 | 250 |
| Mar | 180-220 | 75 | 60 | 100 | 300 | 300 |
| Apr | 0 | 75 | 60 | 175 | 250 | 235 |
| May | 0 | 75 | 60 | 225 | 300 | 285 |
| Jun | 0 | 25 | 15 | 250 | 275 | 265 |
| Jul | 0 | 25 | 15 | 225 | 250 | 240 |
| Aug | 0 | 25 | 15 | 175 | 200 | 190 |
| Sep | 0 | 25 | 15 | 150 | 175 | 165 |

Table S10: Merced River flow requirements driving minimum releases from New Exchequer Reservoir (cfs, left columns). Right two columns (grey) are WEAP operationalizations of Normal and Dry year minimum flows below Crocker-Huffman. According to the requirements, flow below Crocker-Huffman Diversion Dam must equal the greater of the Davis-Grunsky and FERC flows, plus the Cowell Agreement entitlement. See definitions of normal and dry year, and description of forecasting logic, in the text. The Cowell agreement contains provisions in some months for flow reductions based on Merced River daily natural flows, which we have not attempted to include in the modeling given the monthly time step. Davis-Gunsky Flows are operationalized as 200 cfs. October FERC Flows are average values of the given October minimum flows.

|  | **Month (water year)** | | | | | | | | | | | |
| --- | --- | --- | --- | --- | --- | --- | --- | --- | --- | --- | --- | --- |
|  | Oct | Nov | Dec | Jan | Feb | Mar | Apr | May | Jun | Jul | Aug | Sep |
|  |  | | | | | | | | | | | |
|  | Wet Year Flow Requirements (cfs) | | | | | | | | | | | |
|  |  | | | | | | | | | | | |
| **Stanislaus River** | 342 |  |  |  | 684 | 732 | 1361 | 1187 | 538 |  |  |  |
| **Tuolumne River** | 602 |  |  |  | 1080 | 1073 | 1933 | 1870 | 1076 |  |  |  |
| **Merced River** | 260 |  |  |  | 630 | 585 | 1042 | 1008 | 521 |  |  |  |
|  |  |  |  |  |  |  |  |  |  |  |  |  |
|  | Above Normal Year Flow Requirements (cfs) | | | | | | | | | | | |
| **Stanislaus River** | 342 |  |  |  | 666 | 699 | 1160 | 976 | 403 |  |  |  |
| **Tuolumne River** | 602 |  |  |  | 1044 | 1057 | 1630 | 1529 | 823 |  |  |  |
| **Merced River** | 260 |  |  |  | 612 | 569 | 874 | 829 | 403 |  |  |  |
|  |  |  |  |  |  |  |  |  |  |  |  |  |
|  | Below Normal Year Flow Requirements (cfs) | | | | | | | | | | | |
| **Stanislaus River** | 342 |  |  |  | 432 | 472 | 857 | 716 | 269 |  |  |  |
| **Tuolumne River** | 602 |  |  |  | 666 | 699 | 1227 | 1138 | 555 |  |  |  |
| **Merced River** | 260 |  |  |  | 396 | 374 | 655 | 618 | 269 |  |  |  |
|  |  |  |  |  |  |  |  |  |  |  |  |  |
|  | Dry Year Flow Requirements (cfs) | | | | | | | | | | | |
| **Stanislaus River** | 358 |  |  |  | 450 | 472 | 706 | 553 | 235 |  |  |  |
| **Tuolumne River** | 634 |  |  |  | 702 | 699 | 992 | 862 | 454 |  |  |  |
| **Merced River** | 260 |  |  |  | 414 | 374 | 538 | 472 | 218 |  |  |  |
|  |  |  |  |  |  |  |  |  |  |  |  |  |
|  | Critically Dry Year Flow Requirements (cfs) | | | | | | | | | | | |
| **Stanislaus River** | 293 |  |  |  | 162 | 195 | 454 | 374 | 118 |  |  |  |
| **Tuolumne River** | 520 |  |  |  | 270 | 309 | 655 | 585 | 235 |  |  |  |
| **Merced River** | 211 |  |  |  | 162 | 163 | 353 | 325 | 118 |  |  |  |

Table S11: Proxy for estimated D-1641 flow requirements from the Stanislaus, Tuolumne, and Merced Rivers. Monthly required flows (cfs) based on 1922-1992 average unimpaired flows and DWRSIM Study 1995C06F-SWRCB-469 data, using Flow Alternative 5. These tables are based on modeling conducted by DWR using DWRDSM to assess potential contributions of each stream to meeting Vernalis flow requirements as part of the Environmental Impact Report for the 1995 Bay Delta Plan. We used these results as a proxy for required releases in each stream for purposes of meeting D-1641 requirements.

# References for supplemental material

1. United States Bureau of Reclamation (2005) CALSIM II San Joaquin River Model (DRAFT). Sacramento, CA: U.S. Bureau of Reclamation, Mid Pacific Region.

2. Leonardson R, Dale L, Vicuna S, Dracup JA (2005) On the accuracy of streamflow predictions under climate change: Draft Report. Berkeley, CA: California Climate Change Center.

3. Milly PCD, Betancourt J, Falkenmark M, Hirsch RM, Kundzewicz ZW, et al. (2008) Stationarity Is Dead: Whither Water Management? Science 319: 573-574.

4. State Water Resources Control Board (1999) Water Right Decision 1641, In the Matter of: Implementation of Water Quality Objectives for the San Francisco Bay/Sacramento-San Joaquin Delta Estuary; A Petition to Change Points of Diversion of the Central Valley Project and the State Water Project in the Southern Delta; and A Petition to Change Places of Use and Purposes of Use of the Central Valley Project. Sacramento, CA: State Water Resources Control Board, California Resources Agency.

5. State Water Resources Control Board (1995) Water Quality Control Plan for the San Francisco Bay/ Sacramento-San Joaquin Delta Estuary Sacramento, CA: California Environmental Protection Agency. 95-1WR 95-1WR

6. Federal Energy Regulatory Commission (1996) Order Amending License and Dismissing Rehearing Request, Turlock Irrigation District and Modesto Irrigation District, Project Nos. 2299-024 and -031.

7. California Department of Water Resources (2009) Bulletin 120: Water Conditions in California.

8. MBK Engineers (2001) Merced Water Supply Plan Update Final Status Report Exhibit 1: MRSIM. Sacramento, CA: City of Merced, Merced ID, UC Merced.

9. State Water Resources Control Board (1999) Final Environmental Impact Report for Implementation of the 1995 Bay/Delta Water Quality Control Plan: Volume 1. Sacramento, CA: State Water Resources Control Board, California Environmental Protection Agency.

10. State Water Resources Control Board (1999) Final Environmental Impact Report for Implementation of the 1995 Bay/Delta Water Quality Control Plan: Volume 2, Technical Appendices. Sacramento, CA: State Water Resources Control Board, California Environmental Protection Agency.

11. R Core Team (2012) R: A Language and Environment for Statistical Computing version 2.11.0 (2010-04-22). Vienna, Austria: R Foundation for Statistical Computing.

1. Locations of actual DWR snow course locations were used to choose catchments to construct a snow depth index. Catchments comprising the snowpack index were STN_21_3000, STN_16_2500, STN_19_2500, STN_09_2500, STN_05_2000, STN_22_2500. This is a subset of those described below in the Tuolumne Instream Flows section. [↑](#footnote-ref-1)
2. Acre-feet (AF) or variants thereof such as MAF (million acre-feet) or TAF (thousand acre-feet), and cubic feet per second (cfs) are predominantly used in the region both in practice and in legal and regulatory proceedings, and these units are thus retained in this supplemental material. 1 cfs = 0.0283 m^3^/s; 1 AF = 1233.4818 m^3^. [↑](#footnote-ref-2)
3. Sample catchments were chosen as the median elevation catchment within each sub-watershed containing multiple DWR snow course stations, MER_06_3000, MER_05_2500, STN_21_3000, STN_16_2500, STN_19_2500, STN_09_2500, STN_05_2000, STN_22_2500, TUO_13_3000, TUO_10_2500. Snow course locations obtained from CA DWR (Steve Nemeth, DWR, pers. comm.) [↑](#footnote-ref-3)
4. In the Tuolumne River, according to Article 37, water year classifications for FERC-mandated ISF requirements are based on cumulative occurrence of flows, and thus should be updated over time. Here, we have used values based on DWR historical data and have not implemented updating logic. [↑](#footnote-ref-4)
